# Transactions of the American Medical Association, Vol. IX. 1856

**Published:** 1857-02

**Authors:** 


*Transactions of the American Medical Association, Vol. IX.* 1856. Philadelphia: Printed for the Association by T. R. & P. G. Collins.

This is a neatly-printed volume of nine hundred and seven pages, containing, besides the proceedings of the ninth annual meeting and the list of officers and members, the following reports and papers, viz.:—

1. Address of George B. Wood, President of the Association.
2. Report on Deformities after Fractures, by Frank H. Hamilton, M.D.
3. Report on Hydrophobia, by T. W. Blatchford, M.D.
4. Report on the Causes which Impede the Progress of American Medical Literature, by S. D. Gross, M.D.
5. Report of the Committee on Medical Literature, by R. J. Breckenridge, M.D.
6. Report of the Committee on Plans of Organization for State and County Medical Societies, by A. B. Palmer, M.D.
7. Report on the Changes in the Composition and Properties of the Milk of the Human Female, Produced by Menstruation and Pregnancy, by N. S. Davis, M.D.

8. Report on the Sanitary Police of Cities, by James M. Newman, M.D.

9. Report on the Treatment of Cholera Infantum, by A. J. Fuller, M.D.

10. Report on the Use and Effect of Applications of Nitrate of Silver to the Throat, either in General or Local Disease, by Horace Green, M.D.

11. Report on the Best Mode of Making the Patronage of the National Government Tributary to the Honor and Improvement of the Profession, by J. B. Flint, M.D.

12. Report of the Committee on Medical Education, by Wm. H. Anderson, M.D.

13. Report on the Medical Topography of the Eastern Shore of Maryland, by P. Wroth, M.D.

14. History of the Epidemic of Yellow Fever in Charleston, S.C. in 1854, by D. J. Cain, M.D.

15. Report on the Epidemics of Louisiana, Mississippi, Arkansas, and Texas, by E. D. Fenner, M.D.

16. Report on the Meteorology, Mortality, and Sanitary Condition of New Orleans for the years 1854–'55, by E. H. Barton, A.M. M.D.

17. Report on Strychnia; Its Physiological Properties and Chemical Detection, by L. H. Steiner, M.D.

18. Partial Report on a Uniform System of Registration of Births, Marriages, and Deaths, and the Causes of Death, by G. S. Palmer, M.D.

19. Prize Essay, on the Arterial Circulation; Its Physiology and Chief Pathological Relations, by Henry Hartshorne, M.D.

20. Plan of Organization of the American Medical Association.

The address of Dr. Wood, and the proceedings of the last meeting, have already been published in detail in the *Journal*, and will, therefore, be passed over at this time.

The next paper is that of Dr. Hamilton on Deformities after Fractures. It is a continuation of the Report of the same author, made to the Association in May, 1855. The present report fills one hundred and sixty pages, and embraces a consideration of fractures of the scapula, arm, fore-arm, and hand. It is illustrated by four well-executed plates.

The present report of Dr. Hamilton contains an account of one hundred and ninety-three cases of fractures, which, with the results, may be stated in tabular form as follows, viz.:—

| | | | |
|---|---|---|---|
| Fracture of the Scapula...... | 3 | Cure Perfect... 1 | Cure Imperfect... 2 |
| " " Humerus.... | 67 | " " ...24 | " " ...43 |
| " " Radius ...... | 38 | " " ...16 | " " ...22 |
| " " Ulna ......... | 22 | " " ...16 | " " ... 6 |
| " " Radius & Ulna | 41 | " " ...29 | " " ...12 |
| " " Metacarpus | 7 | " " ... 3 | " " ... 4 |
| " " Phalanges.... | 15 | " " ... 9 | " " ... 6 |
| | 193 | 98 | 95 |

From this table it will be seen, that of the whole number of cases reported the result is stated as *imperfect* in almost one-half. By the word imperfect, is meant any result which comes short of complete union, without deformity or shortening of the limb. The number of imperfect results from fractures of the humerus and radius will surprise most of our readers; and so will the difference between the fractures of both bones of the fore-arm, and each of these bones separately. The whole report is highly instructive; but it constitutes only part of an extensive work on fractures which will soon be completed and published in a separate volume. The profession will hail its appearance with much pleasure.

The next paper in the Transactions, is a Report from Drs. Blatchford and Spoor on the subject of Hydrophobia. It embodies a large amount of statistical information in relation to the prevalence of this most dreaded disease, both in this and other countries. One of the leading objects of this report, was to elucidate the question whether there is any truth in the popular notion that the *canine* race are chiefly liable to attacks of rabies during the heat of summer, or what is usually called Dog-Days. After a careful and laborious investigation the Committee has arrived at the conclusion that there is not only no truth in the popular belief about dog-days, but, further, that the hydrophobia both in man and animals has actually occurred more frequently in the cold than the warm months of the year. The practical importance of this investigation is obvious. The idea that the disease attacks dogs chiefly during the five or six hottest weeks of summer, has led to the adoption of municipal regulutions in most of the cities, requiring these animals to be either killed or muzzled during that season, while they are left perfectly at large during all the rest of the year. The entire futility of such regulations will be apparent from the following paragraph and statistical table taken from the report before us:

'Although statistics of rabies go to show that, contrary to popular prejudice, it occurs most frequently in cold countries, and during autumn, winter, and spring, still it appears that of the whole number of cases occurring out of the tropics during the year, *nearly* an equal proportion occurs during each month of the year, from which it may be inferred that the appearance and prevalence of the disease, at particular seasons and in certain localities or regions, are *accidental* and in no way connected with or produced by any thermal or sidereal influence.'

The industrious author of the report has gathered from the United States, England, and France, two hundred and fifty cases of hydrophobia, with the month during which the *bite* took place. Of these, one hundred and twenty-eight were in this country, and one hundred and twenty-two in England and France. The following shows the season of the year in which the bites occurred, viz.:—

| | U.S.A. | England & France. | Total. |
|---|---|---|---|
| *Spring*—March, April, May | 38 | 40 | 78 |
| *Summer*—June, July, August | 28 | 31 | 59 |
| *Autumn*—Sept. Oct. Nov | 30 | 18 | 48 |
| *Winter*—Dec. Jan. Feb | 32 | 33 | 65 |
| | 128 | 122 | 250 |

Of eighty-nine cases in which the period of incubation, or the time between the bite and the active manifestation of the disease was definitely ascertained, the average period was seventy days. In twenty-three cases it was under thirty days, and in six it was over two hundred days.

The report is full of interesting facts, and should be extensively read both in and out of the profession.

(To be Continued.)

